# Recent Advances in Single Fe-Based Nanoagents for Photothermal–Chemodynamic Cancer Therapy

**DOI:** 10.3390/bios12020086

**Published:** 2022-01-31

**Authors:** Li Zhang, Helen Forgham, Ao Shen, Ruirui Qiao, Bing Guo

**Affiliations:** 1 Shenzhen Key Laboratory of Flexible Printed Electronics Technology and School of Science, Harbin Institute of Technology, Shenzhen 518055, China; 1992zhangl@gmail.com; 2 Australian Institute of Bioengineering & Nanotechnology, The University of Queensland, Brisbane, QLD 4072, Australia; h.forgham@uq.edu.au (H.F.); a.shen@uq.net.au (A.S.)

**Keywords:** synergetic performance, photothermal therapy, chemodynamic therapy, second near-infrared biowindow, Fe-based nanoplatforms

## Abstract

Monomodal cancer therapies are often unsatisfactory, leading to suboptimal treatment effects that result in either an inability to stop growth and metastasis or prevent relapse. Thus, synergistic strategies that combine different therapeutic modalities to improve performance have become the new research trend. In this regard, the integration of photothermal therapy (PTT) with chemodynamic therapy (CDT), especially PTT/CDT in the second near-infrared (NIR-II) biowindow, has been demonstrated to be a highly efficient and relatively safe concept. With the rapid development of nanotechnology, nanoparticles can be designed from specific elements, such as Fe, that are equipped with both PTT and CDT therapeutic functions. In this review, we provide an update on the recent advances in Fe-based nanoplatforms for combined PTT/CDT. The perspectives on further improvement of the curative efficiency are described, highlighting the important scientific obstacles that require resolution in order to reach greater heights of clinical success. We hope this review will inspire the interest of researchers in developing novel Fe-based nanomedicines for multifunctional theranostics.

## 1. Introduction

Cancer is one of the most lethal killers and threatens the life of human beings all around the world [1,2,3,4]. Surgery, chemotherapy and radiotherapy are the most routine clinical approaches. Unfortunately, these current treatment modalities are likely to inflict a certain amount of collateral damage to normal tissues because of nonspecific toxicity and high dose treatment/radiation regimens, and regrettably, the damaging effects may last a lifetime [5,6,7,8,9]. Additionally, multidrug resistance and an inability to prevent cancer metastasis during the treatment process may result in poor patient outcomes [10,11]. Therefore, the development of alternative therapeutic strategies is much needed. An emerging cancer therapeutic concept, first proposed by Shi and co-workers in 2016, is chemodynamic therapy (CDT). Since its initial inception, CDT has gone on to attract widespread attention [12,13,14]. CDT uses diverse transition metal ions, such as Fe^2+^, Mn^2+^ and Cu^+^, to catalyze hydrogen peroxide (H_2_O_2_) decomposition within the tumor region. This results in the production of highly reactive and toxic hydroxyl radicals (•OH) in a process known as the chemistry of Fenton or Fenton-like reactions [14,15]. CDT has the potential to be more superior in its approach to treating cancer than current clinical methods because of the following merits: (1) tumor selectivity and weak side effects, (2) no need for external stimuli, (3) tumor microenvironment (TME) modulation and (4) low treatment cost [16,17]. Specifically, CDT is more attractive compared with another kind of reactive oxygen species (ROS)-induced treatment modality, namely, photodynamic therapy (PDT), as traditional PDT is severely restricted by the hypoxic TME and limited penetration depth of the laser (wavelength < 700 nm) [18,19,20]. However, unfavorable catalytic conditions in the TME, including insufficient H_2_O_2_ levels, moderate acidic pH and the overproduction of glutathione (GSH), still impede the treatment effects of CDT for further applications [21,22,23,24,25]. In light of these issues, several methods are currently being adopted to boost CDT efficacy. For instance, copper peroxide (CuO_2_) and calcium peroxide (CaO_2_) nanoparticles are reported to improve CDT by self-supplying H_2_O_2_ [23,26]. Lin et al. [24] constructed a self-reinforcing CDT nanoagent based on MnO_2_ that has both Fenton-like Mn^2+^ delivery and GSH depletion properties. Furthermore, a near-infrared (NIR) light-activated H^+^ release strategy based on upconversion nanoparticles and photoacids is proposed to successfully enhance CDT [27]. Although some progress has been made, it is still difficult for monomodal cancer therapies to achieve satisfactory treatment outcomes, resulting in either an inability to stop growth and metastasis or prevent relapse [28]. Nowadays, researchers are attempting to improve the effects of CDT by shifting their focus from a single CDT to CDT-based combined therapies. These combined approaches include CDT/chemotherapy, CDT/photothermal therapy (PTT), CDT/PDT, CDT/sonodynamic therapy (SDT), CDT/gene therapy and CDT/immunotherapy [29,30,31,32,33,34]. It is hoped that the trend in applying approaches such as these will better target the diversity, complexity and heterogeneity of tumors and the associated TME. Additionally, combining these different therapeutic modalities will result in an improvement to their individual use, which has previously proven to be less than effective in fully eradicating tumors [29,35].

Among the various CDT-combined approaches under investigation, CDT/PTT has shown to be a highly efficient and relatively safe paradigm [36,37]. PTT utilizes diverse photothermal agents (PTAs) to harvest NIR light and rapidly converts the light energy into heat to destroy tumors [38,39]. It is extremely attractive as a cancer treatment modality that proffers various benefits, including non-invasiveness, spatiotemporal controllability, high treatment efficacy and low normal tissue damage [40,41]. PTT can be performed at either NIR-I (700–1000 nm) or NIR-II (1000–1700 nm) biowindow. However, NIR-II light-triggered PTT is a better cancer therapeutic option, as NIR-II light is able to penetrate deeper, possesses a higher maximum permissible exposure (MPE) (1 W cm^−2^ for 1064 nm, 0.72 W cm^−2^ for 980 nm and 0.33 W cm^−2^ for 808 nm) and leads to less tissue attenuation [42,43,44]. With these in mind, researchers have looked toward developing superlative NIR-II PTAs from nanomaterials that are both inorganic (such as gold nanoparticles and metal chalcogenides) and organic (such as conjugated small molecules and polymers) [42,45,46,47]. Combining PTT and CDT is a clever proposal because, in addition to directly ablating cancer cells, the photothermal effects during PTT can also accelerate the reaction rate of the Fenton-based process and improve CDT by generating more •OH. Specifically, kinetics studies have revealed that the Fenton reaction rate could achieve fourfold augmentation when an area was heated from 20 to 50 °C [48].

Several studies have reviewed the design and synthesis of photothermal–chemodynamic theranostic nanoplatforms that have been fabricated by covalently or physically integrating multiple different therapeutic subunits. The manufacture of these nanoplatforms is routinely a complicated synthetic process, resulting in poor reproducibility and inherent instability [26,27,28]. Therefore, it is of great significance to develop a single unit nanoagent that possesses both PTT- and CDT-related features. Fe is the most abundant trace element in human beings, making them generally safer and more biocompatible than some other nanomaterials [49,50]. In addition, Fe-based nanomaterials display other in-built characteristics, which have garnered a lot of attention in both cancer diagnosis and the therapeutics field. These fundamental attributes include an inherent magnetic property and excellent stability. Moreover, they readily promote the induction of catalytic activities and contain NIR-light-responsive properties [51,52,53]. In this work, we summarize the recent advances of single Fe-based nanoagents for combined PTT/CDT. The current challenges and opportunities for the design and clinical application of these state-of-the-art nanoplatforms are also described.

## 2. Nanoplatforms for PTT/CDT in the NIR-I Biowindow

To date, various Fe-based nanomaterials, such as iron sulfide (Fe_3_S_4_), copper iron sulfide (CuFeS_2_), Fe-doped nanoagents and Fe-complexes, have been investigated for synergistic PTT/CDT. To ensure efficacy, nanoplatforms combining PTT and CDT should display low toxicity, good biocompatibility, be of an appropriate size for tumor accumulation and exhibit excellent light absorption/light-to-heat conversion and catalytic potential [54,55]. Moreover, it was reported that nanomaterials with a particle size less than 6 nm could undergo rapid renal clearance; therefore, avoiding excessive accumulation in the mononuclear phagocyte system and preventing notable toxicity [56,57]. In this respect, a transformed nanoplatform based on polyvinyl pyrrolidone (PVP)-coated Fe_3_S_4_ (PVP-Fe_3_S_4_) tetragonal nanosheets was developed by Guan et al. [58]. The PVP-Fe_3_S_4_ nanosheets possessed strong absorption in the NIR region, an excellent photothermal conversion efficiency (PCE = 64.3% at 915 nm laser) and good *T*_2_-weighted magnetic resonance (MR) imaging performance (transverse relaxivity, *r*_2_ = 71.3 mM^−1^ s^−1^). In addition, the localized heat produced by PTT was able to promote the initiation of a Fenton reaction by catalyzing endogenous H_2_O_2_ to produce supplementary •OH that could, in return, help suppress tumor growth and recurrence. More interestingly, contributing to the oxidation process and reductive dissolution/recrystallization process, the PVP-Fe_3_S_4_ nanosheets with an edge length of 120 ± 18 nm gradually released Fe^3+^ and transformed into small particles of ~5 nm in diameter in the normal physiological environment over three weeks. The presence of H_2_O_2_ shortened the transition time and the same transformed product could be generated in the TME. These were then effectively excreted from the body after exerting their therapeutic effect.

**Figure 1:**
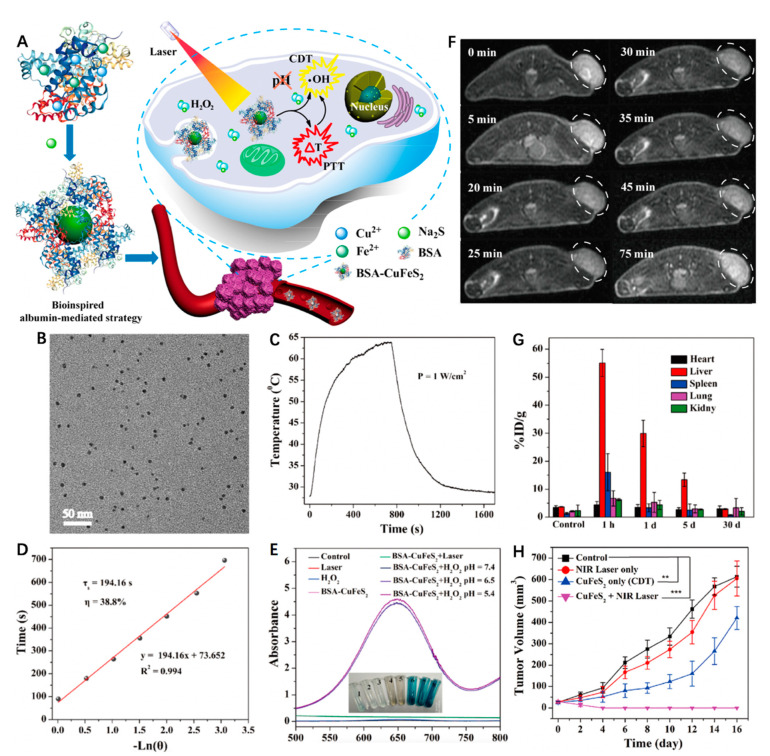
(**A**) Schematic illustrating the synthesis of BSA-CuFeS_2_ nanoparticles and their applications for synergetic pH-independent CDT/PTT. (**B**) Transmission electron microscope (TEM) image of the BSA-CuFeS_2_ NPs. (**C**) Photothermal effect of the BSA-CuFeS_2_ aqueous suspensions ([Cu] = 100 ppm) under irradiation, and then the laser was shut off (808 nm laser, 1 W cm^−2^). (**D**) Calculation of the time constant (τ_s_) and PCE. (**E**) UV−vis spectra of the 3,3,5,5-tetramethylbenzidine (TMB) aqueous with or without H_2_O_2_ or BSA-CuFeS_2_ at varying pH values. BSA-CuFeS_2_ with H_2_O_2_ could catalyze the reaction of TMB to cause a blue color reaction at varying pH conditions with maximum absorbance at 652 nm. Inset: corresponding different color reactions of samples. (**F**) *T*_2_-weighted MR images of mice bearing transplanted 4T1 tumors injected intravenously with BSA-CuFeS_2_ (15 mg kg^−1^). (**G**) Biodistribution of Cu in the major organs at varying times. (**H**) Tumor growth volume curves after different treatments. Reprinted with permission from Ref. [60]. Copyright 2019, American Chemical Society.

Copper sulfide (CuS) nanoparticles were shown to possess superior photoabsorption properties in the NIR region [59]. Therefore, the introduction of a CuS component to an Fe-based matrix is expected to enhance the curative effects of PTT/CDT. Chen et al. [60] employed a facile aqueous biomineralization strategy to synthesize ultrasmall bovine serum albumin (BSA)-modified chalcopyrite (BSA-CuFeS_2_) nanoparticles with a diameter around 4.9 nm and good dispersity, as well as biocompatibility (Figure 1A,B). Remarkably different from the classical pH-dependent Fenton reaction (i.e., generally energetic in a narrow pH range (e.g., pH = 3–4)), BSA-CuFeS_2_ was able to produce a comparable amount of •OH in varying pH conditions (7.4, 6.5 and 5.4) for CDT (Figure 1E). This pH-independent Fenton-like reaction of the BSA-CuFeS_2_, similar to Cu-based Fenton-like reactions, could work over a wide pH range, including neutral pH conditions. Combined with good photothermal effects (PCE = 38.8% at 808 nm), BSA-CuFeS_2_ produced a synergistic effect between PTT and CDT (Figure 1C,D). In contrast to the tumor volumes of the control group (~600 mm^3^), the tumor growth was apparently inhibited in the single CDT group, while the PTT/PDT group showed thorough tumor ablation after the treatment (Figure 1H). In addition, BSA-CuFeS_2_ was also an efficient *T*_2_-weighted MR imaging contrast agent (*r*_2_ = 5.06 mM^−1^ s^−1^) that caused a remarkable and quick decrease in the MR signal at the tumor site (Figure 1F). In order to identify the long-term biodistribution and clearance, the Cu concentrations in solubilized main organs were measured using inductively coupled plasma atomic emission spectrometry (ICP-AES) (Figure 1G). It was clear that the liver, spleen and kidney had a significant Cu uptake at 1 h post injection. After 5 days, the Cu concentration significantly decreased to 15 ± 4.7 and 3.3% ID/g in the liver and spleen, respectively, suggesting the body-clearable nature of BSA-CuFeS_2_ NPs. Notably, the Cu concentration in the kidney rapidly decreased and closed to the level of the control group after 5 days, revealing that the ultrasmall size of BSA-CuFeS_2_ NPs could lead to renal clearance.

**Figure 2:**
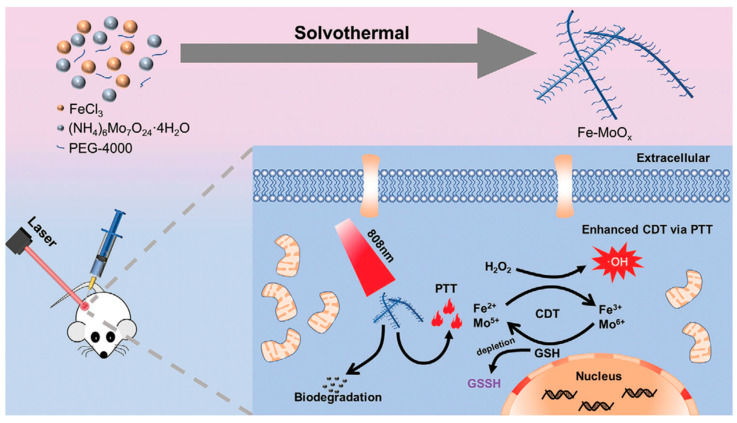
Schematic depicting the synthesis of FMO nanowires and demonstrating FMO for photothermal enhanced CDT and GSH-depleted amplified CDT. Reprinted with permission from Ref. [61]. Copyright 2021, Wiley-VCH Verlag GmbH & Co. KGaA, Weinheim.

Chen et al. [61] also designed and characterized state-of-the-art biodegradable Fe-doped MoOx (FMO) nanowires using a one-step solvothermal method and then modified them to incorporate polyethylene glycol-4000 (PEG-4000). FMO, as an anti-tumor nanoagent, possessed a high PCE (48.5% at 808 nm) and demonstrated excellent magnetic performance for *T*_1_-weighted MR imaging due to the unpaired electrons of the Fe^3+^ ions (longitudinal relaxivity, *r*_1_ = 1.4107 mM^−1^ s^−1^). Moreover, FMO was capable of effectively catalyzing the decomposition of H_2_O_2_ to form •OH for enhanced CDT, accompanied by the consumption of GSH (Fe^3+^/Fe^2+^ and Mo^5+^/Mo^6+^ reduction). Importantly, FMO presented with excellent pH-dependent degradation, which is a behavior that encouraged rapid degradation at a physiologically relevant pH whilst also remaining relative stability at an acidic pH. The findings, therefore, suggested that FMO was highly biodegradable and would not promote long-term toxicity (Figure 2).

**Figure 3:**
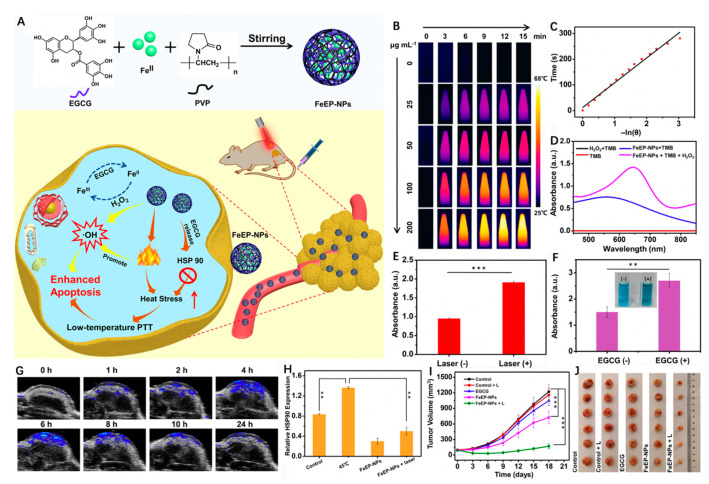
(**A**) Schematic detailing synthesis of the FeEP nanoplatform and illustrating its applications for mild PTT-enhanced CDT to promote tumor ablation. (**B**) Thermal images of FeEP with different concentrations (µg mL^−1^) under 808 nm laser irradiation (1 W cm^−2^, 15 min). (**C**) Fitted linear relationship between −ln θ and time. (**D**) UV-vis absorbance spectra of tetramethylbenzidine (TMB), H_2_O_2_ + TMB, FeEP + TMB and FeEP + H_2_O_2_ + TMB. (**E**) UV-vis absorbance of TMB at the wavelength of 652 nm after being co-incubated with FeEP and H_2_O_2_ together with and without laser irradiation. (**F**) UV-vis absorbance of TMB at the wavelength of 652 nm after incubation with Fe II + H_2_O_2_ or Fe II + H_2_O_2_ + EGCG. (*) *p* < 0.05, (**) *p* < 0.01 and (***) *p* < 0.001. (**G**) PA images of the tumor region from 4T1 tumor-bearing mice before and after intravenous injection of FeEP. (**H**) The relative HSP 90 expression level in 4T1 cells with different treatments. (**I**) The growth curves of tumor volumes of the mice after various treatments. (*) *p* < 0.05, (**) *p* < 0.01 and (***) *p* < 0.001. (**J**) Digital photography of the excised tumors from different mouse groups. Reprinted with permission from Ref. [63]. Copyright 2021, Elsevier Inc.

Fe-complexes also show great promise for synergistic PTT/CDT [62]. Based on the coordinated interaction between Fe^2+^ and (-)-epigallocatechin gallate (EGCG), the major polyphenol found in green tea, Yu et al. [63] utilized a one-pot self-assembly method to fabricate a new multifunctional nanoagent (FeEP) (Figure 3). The FeEP nanoparticles could effectively generate a toxic •OH via Fenton reaction for CDT. Intriguingly, the specific binding between Fe^2+^ and EGCG equipped the nanoparticles with an intensive NIR absorption capability, good photoacoustic (PA) imaging performance and photothermal conversion ability (PCE = 33.6% at 808 nm), activating photothermal-enhanced CDT when the FeEP nanoparticles were exposed to laser irradiation. Notably, the partial release of EGCG was able to accelerate the conversion of Fe^3+^/Fe^2+^ to amplify the •OH production and further promote CDT. Simultaneously, the release of EGCG also resulted in the downregulation of intracellular heat shock protein 90 (HSP 90) expression, which improved the PTT effect. As expected, both in vitro and in vivo experimental data demonstrated that tumor growth was inhibited more efficiently in the FeEP plus laser group due to the low-temperature PTT-potentiated CDT effects. A similar study was also reported by Liu et al. [64]. In their study, Fe^2+^ was coordinated to baicalein (Ba), a natural polyphenol extracted from medical herbs, using a solution-based wet chemical method. After PEG modification, the Fe–BaP showed a desirable PCE of 45.6% upon exposure to 808 nm laser irradiation that synergistically promoted •OH production. Moreover, the Fe^3+^ oxidized by H_2_O_2_ promoted a ripple effect by reacting with the released baicalein to form Fe^2+^, producing high Fenton activity, which subsequently improved the efficiency of the CDT.

## 3. Nanoplatforms for PTT/CDT in the NIR-II Biowindow

As it stands, inadequate penetration depths and the low MPE of commonly utilized 808 nm lasers restrict the application of PTT/CDT. Based on standards set by the Food and Drug Administration (FDA), the clinically approved MPE for skin is 0.33 W cm^−2^ for an 808 nm laser and 1.0 W cm^−2^ for a 1064 nm laser [65]. Therefore, research into the development of more effective PTT/CDT has shifted toward identifying nanoplatforms that can take advantage of and increase the therapeutic potential of the deeper penetrating NIR-II biowindow [66,67]. Iron sulfide/phosphide (FeS_2_, Fe_2_P and FePS_3_), copper iron sulfide (Cu_5_FeS_4_ and CuFe_2_S_3_) and an Fe-doped nanoagent for NIR-II PTT/CDT are of much interest in this respect.

### 3.1. Iron Sulphide/Phosphide Nanoparticles

**Figure 4:**
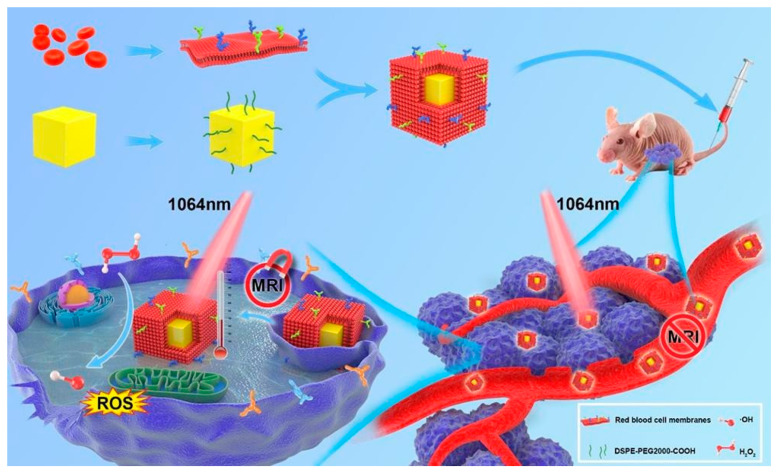
Schematic illustrating the fabrication and anti-tumor effect of FeS_2_@RBCs in vivo. With RBCs coating, FeS_2_@RBCs exhibited prolonged blood circulation, leading to improved tumor accumulation. FeS_2_@RBCs showed TME-enhanced MRI after reacting with H_2_O_2_ in tumor regions for imaging-guided PTT. With an FDA-approved 1064 nm laser, FeS_2_@RBCs achieved effective PTT, which significantly augmented the CDT effects for tumor synergetic therapy. The growth of tumors could be significantly inhibited by a clinically approved NIR-II laser. Reprinted with permission from Ref. [68]. Copyright 2020, Elsevier Inc.

A rationally designed red blood cell membrane (RBC)-coated FeS_2_ (FeS_2_@RBCs) nanoplatform was recently reported by She et al. [68] (Figure 4). The preparation procedure included the fabrication of FeS_2_-PEG, preparation of RBCs vesicles and fusion of RBCs on the surface of FeS_2_-PEG. According to the dynamic light scattering (DLS) results, the hydrodynamic size of FeS_2_@RBCs was measured to be 185.2 nm, which was slightly larger than that of FeS_2_-PEG (168.3 nm) owing to the RBCs coating. The obtained FeS_2_@RBCs possessed strong optical absorption within the NIR-II area, resulting in effective PTT-augmented CDT. Due to the specific surface properties of RBCs, the nanoplatform could effectively enter into the 4T1 cells with the extension of incubation time. Compared with the FeS_2_-PEG group (4.2% ID/g), the FeS_2_@RBCs group achieved significantly higher tumor accumulation with 8.7% ID/g at 6 h post injection. After 24 h post injection, the tumor accumulation of FeS_2_@RBCs was still higher than that of FeS_2_-PEG, which was attributed to the superior blood circulation. Notably, FeS_2_@RBCs exhibited obvious lower uptake by the liver and spleen in contrast to FeS_2_-PEG, revealing its immunity evasion capabilities. These results culminated in an enhancement in tumor accumulation and improved PTT (PCE = 30.2%) under 1064 nm laser irradiation. Moreover, CDT efficiency was improved through the increased generation of •OH that was brought about by the elevated temperature at the tumor site. This was demonstrated through the peroxidation of lipid, which was identifiable through the cell death pathway of CDT. In addition, FeS_2_@RBCs exhibited an elegant self-enhanced MR imaging ability after H_2_O_2_ treatment that could be used to guide the therapy. As anticipated, subcutaneous 4T1 breast tumor growth was remarkably inhibited with negligible side effects arising from the combined NIR-II PTT and CDT.

**Figure 5:**
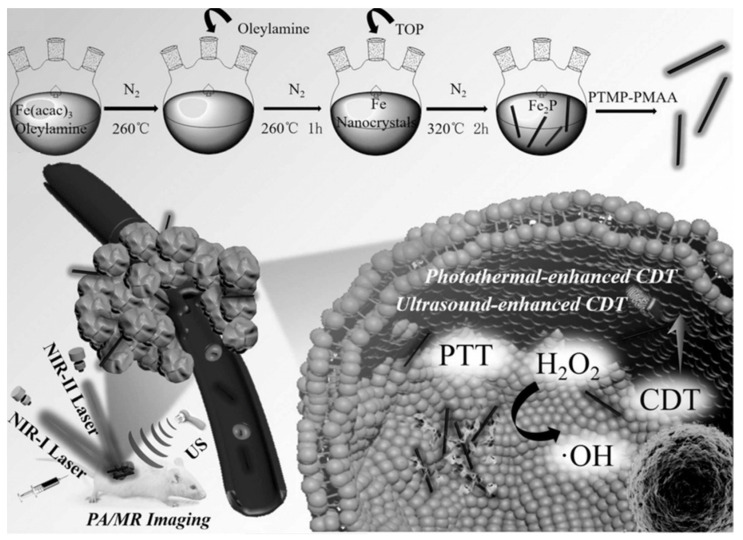
Schematic illustration of the synthesis of FP NRs and their applications for US/PT-enhanced CDT and PTT in the NIR I/II window. PTMP stands for pentaerythritol tetrakis 3-mercaptopropionate and TOP stands for trioctylphosphine. Reprinted with permission from Ref. [69]. Copyright 2019, Wiley-VCH Verlag GmbH & Co. KGaA, Weinheim.

Liu et al. [69] fabricated novel one-dimensional (1D) Fe_2_P nanorods (NRs) that were fitted with a trithiol-terminated poly(methacrylic acid) (PTMP-PMAA) modification (FP NRs) in order to enhance hydrophilicity and biocompatibility. The FP NRs exhibited excellent photothermal effects (PCE = 56.6% at 1064 nm) and considerable Fenton effects. Interestingly, the Fenton reaction efficiency could be dramatically improved using PTT and with the assistance of ultrasound (US). Moreover, the FP NRs had a high impairment ratio, regardless of the limitations of depth and low-dose laser intensity. Additionally, an intrinsic photothermal conversion ability and magnetic property presented FP NRs as a noteworthy and promising new tool for enhancing dual-modal PA and MR imaging (*r*_2_ = 277.79 mM^−1^ s^−1^). Therapeutically, in vitro and in vivo experimental results demonstrated that FP NRs were effective as a treatment method for tumor ablation owing to the synergistic effect of NIR-II PTT and photothermal/US co-enhanced CDT (Figure 5).

**Figure 6:**
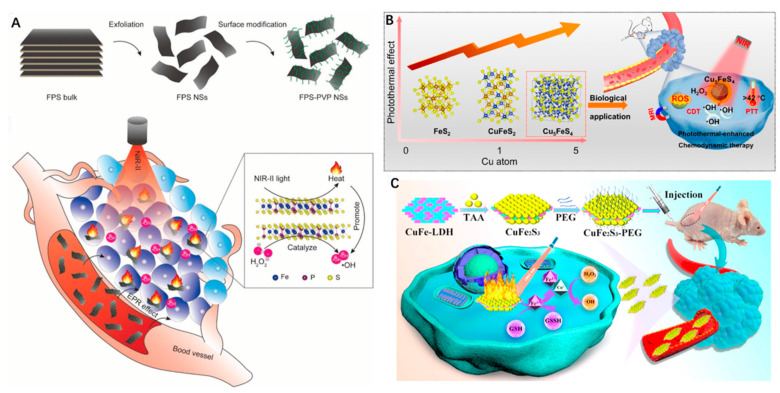
(**A**) Scheme of synthetic process of FPS-PVP nanosheets and their applications for synergistic CDT and PTT in the NIR-II biowindow. Inset is the ball-and-stick model of FPS. Reprinted with permission from Ref. [70]. Copyright 2020, Wiley-VCH Verlag GmbH & Co. KGaA, Weinheim. (**B**) The photoabsorption and photothermal effect of Cu_x_Fe_y_S_z_ nanomaterials can be tuned by changing the Cu/Fe ratios, and Cu_5_FeS_4_ can be used as an efficient ‘‘one-for-all” type agent for MR imaging-guided photothermal-enhanced CDT. Reprinted with permission from Ref. [73]. Copyright 2021, Elsevier Inc. (**C**) Schematic illustration of the preparation of CuFe_2_S_3_-PEG nanosheets for efficient NIR-II PTT and CDT. Reprinted with permission from Ref. [74]. Copyright 2021, Elsevier Inc.

Zhang et al. [70] reported a new 2D nanoplatform based on biocompatible FePS_3_ (denoted as FPS) nanosheets for the first time. The FPS nanosheets were initially obtained via a facile liquid exfoliation from bulk material. Further surface modification was performed in order to attach PVP (FPS-PVP), which improved water dispersibility and stability. The FPS-PVP nanosheets possessed a high PCE of 43.3% when exposed to a 1064 nm laser for highly efficient PTT. Concomitantly, the nanosheets exhibited an excellent Fenton catalytic activity for CDT that was ascribed to the highly targeted surface area and a therapeutic efficiency boosted under laser irradiation. The resultant effect was tumor elimination without relapse (Figure 6A).

### 3.2. Copper Iron Sulfide Nanoparticles

In recent years, CuS nanoparticles have also been widely explored for PTT within the NIR-II biowindow. Therefore, it also stands to reason that copper, iron and sulphide mixed nanoparticles with differing concentrations ratios of each element (Cu_x_Fe_y_S_z_) could hold great promise when aiming to combine NIR-II PTT with CDT [71,72]. A series of Cu_x_Fe_y_S_z_ nanoparticles were prepared by Wang et al. [73] through increasing the ratios of Cu:Fe from 0:1 to 1:1 and 5:1. Using the increasing ratios of Cu:Fe, the authors systematically investigated how the composition and structural transformation affected both the optical and photothermal properties of these nanoparticles (FeS_2_, ~24 nm; CuFeS_2_, ~20 nm; Cu_5_FeS_4_, ~22 nm). Interestingly, the authors observed an evident red-shift in the localized surface plasmon resonances characteristic peak of Cu_5_FeS_4_ nanoparticles and the Cu_5_FeS_4_ also exhibited the highest PCE (45.9%), in contrast to the FeS_2_ (24.4%) and CuFeS_2_ (36.6%) when exposed to a 1064 nm laser. The Cu_5_FeS_4_ nanoparticles displayed the desired photothermal-enhanced Fenton effects necessary to form abundant •OH under different pH conditions for enhanced CDT. Furthermore, the Cu_5_FeS_4_ nanoparticles were also able to serve as an efficient *T*_2_-weighted MR imaging contrast agent due to their excellent magnetic characteristics. At 2 h post injection, the signal intensity decreased sharply and then showed an increase, identifying the best time point for the maximum accumulation of Cu_5_FeS_4_ nanoparticles within the tumor. Importantly, a remarkable shrinkage in tumor size was noticed in the Cu_5_FeS_4_ + 1064 nm laser groups after 18 days of treatment, culminating in a 100% survival rate after 40 days. In addition, the treated mice were sacrificed on the 1st, 9th and 18th days post injection, and the main organs were obtained to investigate the biodistribution of Cu_5_FeS_4_. Notably, the Cu ions were mainly accumulated in the liver (23.5% ID/g) and spleen (16.4% ID/g), which are major organs of the reticuloendothelial system (RES). Along with the extension of time, the concentration of Cu showed a decreasing trend and went down to 4.3% ID/g in the liver and 2.0% ID/g in the spleen on the 18th day, illustrating that most of the Cu in the organs was excreted, signifying relatively low retention. Collectively, the Cu_5_FeS_4_ nanoparticles were proven to be an efficient all-in-one nanoplatform for MR imaging-guided NIR-II PTT and CDT (Figure 6B).

Sulfurization of ultrathin CuFe layered double hydroxide (LDH) nanosheets was performed by Wang et al. [74] using a simple hydrothermal method. After PEG modification, the prepared ultrathin 2D CuFe_2_S_3_ nanosheets (a lateral size of ~63 nm and an average thickness of ~1.5 nm) were subsequently tested for the synergistic application of NIR-II PTT and CDT (Figure 6C). The CuFe_2_S_3_-PEG showed broadband optical absorption in the NIR-II region, with a PCE of ~55.86% at 1064 nm. The CuFe_2_S_3_-PEG nanosheets were also shown to react with the overproduced GSH in TME to release Fe^2+^ and Cu^+^, thus triggering Fenton and Fenton-like reactions that generated plentiful •OH for CDT. Moreover, the hyperthermia result of the PTT was able to further promote the Fenton-based reactions. Both in vitro and in vivo experiments confirmed a distinctively efficient PTT/CDT, as demonstrated through significant HepG2 cells death and antitumor effect following CuFe_2_S_3_-PEG plus laser treatment. Consequently, this work identified an elegant strategy that integrates NIR-II PTT and PTT/GSH that is responsive CDT within a single nanoplatform for use as a highly effective cancer therapy.

### 3.3. Iron-Doped Inorganic Nanoparticles

**Figure 7:**
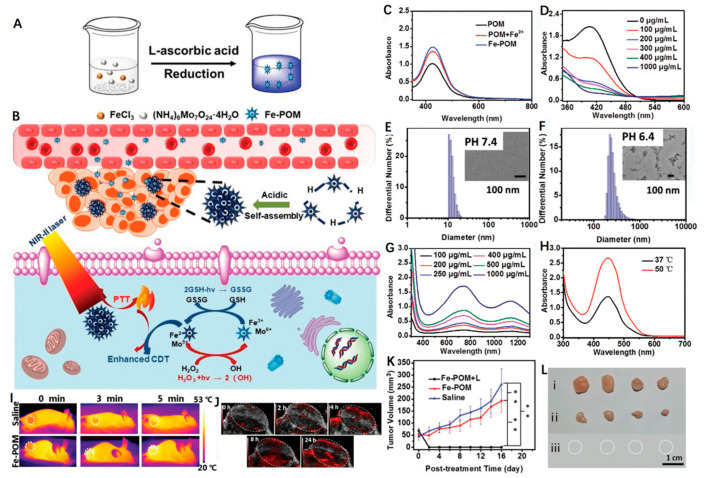
(**A**) A schematic illustrating the fabrication of acid-aggregated Fe-POM cluster. (**B**) Scheme showing Fe-POM for PTT-enhanced CDT in the NIR-II window. (**C**) UV-vis absorption spectra of o-phenylenediamine (OPD) changes by POM, POM + Fe^2+^ and Fe-POM. (**D**) GSH depletion under the reduction of different concentrations of Fe-POM (100, 200, 300, 400, 1000 μg mL^−1^). DLS curves and TEM images of Fe-POM clusters at (**E**) pH 7.4 and (**F**) pH 6.4. (**G**) UV-vis spectra of Fe-POM dispersions at various concentrations. (**H**) UV-vis absorption spectra of OPD incubated with Fe-POM with or without laser irradiation. (**I**) Thermal images of mice injected with saline or Fe-POM (1060 nm laser, 1 W cm^−2^), tumor site with dot line circled. (**J**) In vivo PA images of tumor-bearing mice. (**K**) Tumor volumes in mice received three different groups. *n* = 4, mean ± SD. (**) < 0.01. (**L**) Representative photos of tumors after therapy (after 16 days of treatment). Reprinted with permission from Ref. [76]. Copyright 2020, Wiley-VCH Verlag GmbH & Co. KGaA, Weinheim.

Doping using transition metals ions can significantly improve the structural and optical properties and chemical composition of inorganic nanoparticles [75]. Shi et al. [76] successfully fabricated Fe-doped Mo-based polyoxometalate (Fe-POM) clusters and applied them as a smart theranostic agent for tumor-acidity-specific, PTT-reinforced CDT in the NIR-II biowindow (Figure 7A,B). The presence of Fe^2+^ and Mo^5+^ equipped the Fe-POM clusters with the ability to work as an ideal Fenton agent, catalyzing H_2_O_2_ to generate toxic •OH (Figure 7C). In addition to generating •OH, the Fenton-based reactions also facilitated the formation of Fe^3+^ and Mo^6+^ via Fe^2+^ and Mo^5+^ oxidation, which further participated in the reaction with GSH to produce glutathione disulfide (GSSG) and Fe^2+^/Mo^5+^ (Figure 7D). The initiated cyclic redox reaction led to a continuous decrease in GSH, thus eliminating the cellular antioxidant defense system and, overall, enhancing the CDT effect. Interestingly, when incubated in a neutral medium (pH 7.4), Fe-POM was highly uniform with an ultra-small diameter of 12.9 nm, as revealed by the TEM images and DLS measurement (Figure 7E). However, when the pH of the medium decreased to 6.4, the clusters could aggregate into larger assemblies with a diameter of 271.1 nm within about 4 h and retain their stability (Figure 7F). This phenomenon might be caused by the hydrogen bond formation due to acid-induced protonation, where such larger nanostructures displayed a stronger NIR-II absorbance for PTT (Figure 7G). In vitro, Fe-POM exhibited a high PCE (51.4% at 1060 nm) that killed cancer cells and caused a stimulatory effect on CDT (Figure 7H). In vivo, PA imaging identified a marked accumulation of Fe-POM in tumors that was attributed to their ability to self-assemble in an acidic TME setting (Figure 7I,J). After an Fe-POM plus laser treatment, significant inhibition of tumor growth was achieved due to the synergistically combined PTT/CDT, without any apparent side effects (Figure 7K,L). In summary, this work showcased the potential of all-in-one easy-to-synthesize clusters that promote GSH consumption and independently commit to acidic TME-responsive self-assembly and PA imaging-guided NIR-II PTT/CDT. These notable characteristics have endowed Fe-POM with tremendous potential for use within the cancer nanotheragnostic field.

## 4. Perspectives and Conclusions

To compensate for the unsatisfactory treatment effects of single PTT or CDT, the design and manufacture of a range of diverse nanoplatforms that combine these two treatment modalities have garnered considerable attention over recent years. In this short review, we described a summary of the latest and most promising Fe-based nanoagents for treating various types of tumors (Table 1). These include PVP-Fe_3_S_4_, BSA-CuFeS_2_, Fe-doped MoO_x_, Fe^2+^-EGCG and Fe^2+^-baicalein complexes for NIR-I PTT/CDT and FeS_2_@RBCs, Fe_2_P, FePS_3_, Cu_5_FeS_4_, CuFe_2_S_3_ and Fe-POM for NIR-II PTT/CDT. Due to intrinsic photothermal and magnetic properties, Fe-based nanoagents also boast other diagnostic abilities, namely, PA and MR imaging. All things considered, it is the remarkable and rapid ongoing development within the nanotechnology field that has made this multimodal therapeutic paradigm a real possibility for clinical application. However, there are several issues that remain and that must be resolved prior to effective translation.

Safety is regarded as one of the most important concerns surrounding the application of nanomedicines. Ideal PTT/CDT nanoagents should be non-toxic in the absence of NIR light radiation and H_2_O_2_. To help minimize the toxicity, the above-mentioned studies have incorporated polymers (such as PEG, PVP and PTMP-PMMA), BSA and RBCs to improve the physiological stability, biocompatibility and blood-circulation time, but the lack of targeting capabilities still results in unsatisfactory tumor accumulation. Therefore, cancer-specific units, such as folic acid (FA), hyaluronic acid (HA) and arginine-glycine-aspartic acid (RGD) peptide, should be functionalized on the next-generation Fe-based nanoagents [77,78,79]. Even though the development of biodegradable and clearable Fe-based nanoagents is encouraging, additional research is still needed to fully investigate the short- and long-term safety of these promising nanoagents. To meet all the prerequisite clinical requirements, new types of nanomaterials that possess excellent photostability and PCE are also being explored. These new nanomaterials, together with adjustments to nanoparticle size and shape, alongside heterogeneous ion doping, were shown to be viable options. Since NIR-II-laser-triggered therapy is superior to NIR-I in terms of penetration depth and MPE, the next generation of nanoagents should primarily be focused toward the production of intensive NIR-II absorption. Or perhaps we should even be attempting to extend beyond 1064 nm to longer wavelengths.

Another strategy is to take multi-component transition metals and integrate them into the Fe-based nanoagent, which, as demonstrated, results in the depletion of intratumoral GSH and simultaneously generates abundant ions to conduct efficient Fenton-based reactions that boost CDT. On a slightly different tangent, recent studies have reported that the photothermal-enriched production of ROS can effectively trigger immunogenic cell death (ICD), which is significant enough to prompt systemic anti-tumor immunity resulting in complete tumor eradication post-treatment [80,81]. These compelling findings warrant future studies that investigate the potential synergistic effects between NIR-II PTT/CDT and immunotherapy. Lastly, the ability to design and manufacture simple and reproducible nanoagents that can be attuned to mass production by green and facile methods is a current limitation that must be resolved in order for them to reach clinical applications. With the continuing development of various multi-disciplinary fields and the considerable research being applied to this area, we anticipate that Fe-based nanoagents will quickly be a leader in the field of cancer nanotheranostics and that soon we will see an Fe-based nanoagent being translated from bench to bedside.

## Figures and Tables

**Table 1:** Summary of different Fe-based nanoagents for cancer therapy.

Material	PCE	Laser Wavelength	Tumor Model	Reference
PVP-Fe_3_S_4_	63.4%	915 nm	HeLa tumor	[58]
BSA-CuFeS_2_	38.8%	808 nm	–	[60]
FMO	48.5%	808 nm	HeLa tumor	[61]
FeEP	33.6%	808 nm	4T1 tumor	[63]
Fe–BaP	45.6%	808 nm	4T1 tumor	[64]
FeS_2_@RBCs	30.2%	1064 nm	4T1 tumor	[68]
FP NRs	56.6%	1064 nm	U14 tumor	[69]
FPS-PVP	43.3%	1064 nm	HeLa tumor	[70]
Cu_5_FeS_4_	45.9%	1064 nm	4T1 tumor	[73]
CuFe_2_S_3_-PEG	~55.86%	1064 nm	HepG2 tumor	[74]
Fe-POM	51.4%	1060 nm	HeLa tumor	[76]

## Data Availability

Not applicable.
